# Modeling integrin and plasma-polymerized pyrrole interactions: chemical diversity relevance for cell regeneration

**DOI:** 10.1038/s41598-019-43286-4

**Published:** 2019-05-07

**Authors:** Iris N. Serratos, Roberto Olayo, César Millán-Pacheco, Juan Morales-Corona, Jonathan Osiris Vicente-Escobar, Ana María Soto-Estrada, José Gilberto Córdoba-Herrera, Omar Uribe, Teresa Gómez-Quintero, Miguel Ángel Arroyo-Ornelas, Rafael Godínez-Fernández

**Affiliations:** 1 Departamento de Química, Universidad Autónoma Metropolitana-Iztapalapa, Ciudad de México, Mexico; 2 Departamento de Física, Universidad Autónoma Metropolitana-Iztapalapa, Ciudad de México, Mexico; 3 Facultad de Farmacia, Universidad Autónoma del Estado de Morelos, Morelos, Mexico; 4 Departamento de Ingeniería Eléctrica, Universidad Autónoma Metropolitana-Iztapalapa, Ciudad de México, Mexico

**Keywords:** Cheminformatics, Computational methods

## Abstract

Protein-engineered biomaterials represent a powerful approach to increase biofunctional activity like tissue repair and celular proliferation. Among these materials, integrins and the development of their specific interactions with plasma-polymerized pyrrole (PPPy) are promising biomaterial for tissue regeneration. In this paper, we studied the molecular recognition in the active site of three integrins (α5β1, αvβ3 and αIIbβ3) with PPPy using the structure proposed by Kumar *et al*. PPPy molecule has three sites to incorporate different species, we worked mainly with the functional groups, –NH_2_ and –OH groups according to our IR spectroscopic results. We carried out docking studies to find the better conformational couplings and to determine electrostatic (ΔG_elec_) and non-electrostatic (ΔG_non-elec_) contributions to the binding free energy (ΔG_b_) of these complexes we used Adaptive Poisson-Bolztmann program (APBS). Our results indicated that when incorporating -1H-azirine, -NH_2_ or –OH group in PPPy structure, interactions with integrins were favorable, as indicated by correspondent ΔG_b_ values. These interactions were mainly triggered by Coulomb interactions, an important term in the electrostatic component. Furthermore, our studies suggest that some residues of integrins α5β1, αvβ3 and αIIbβ3 like aspartates are important for the binding to PPPy structures. Detailed interactions between integrin α5β1 and PPPy structures were revealed by molecular dynamics simulations. We used this particular integrin structure because of its favorable ΔG_b_ as well as its major cellular receptor for the extracellular matrix protein fibronectin. Clustering analysis allowed us to carry out focused docking studies and to determine the time evolution of the ΔG_b_ values. By incorporating -NH_2_ into PPPy structure, ΔG_b_ values were very favorable during the course of the dynamics simulations by the establishment of hydrogen bonds with **Asn224** and/or**Asp227** residues, which are part of the integrin α5β1 pocket. However, for the integrin α5β1-PPPy-1H-azirine complex and the rest of the functional groups, the ΔG_b_ values were less favorable, although PPPy was found at a distance of less than 5 Å from the active site residues. This work is complementary to the previous studies made employing PPPy nanoparticles for a variety of tissue engineering applications, and were done to enlighten the role played by the amino group of the PPPy in its integrin recognition process.

## Introduction

Nanoparticles surface can modúlate cellular responses such as tissue regeneration. The interactions established between cells and nanoparticles or other materials are carried out mainly via adhesion proteins including integrins because of their different **cellular** functions.

Integrins are heterodimeric proteins, constituted by alpha and beta subunits, which are connected non-covalently and can also bind cations as Ca^2+^ or Mg^2+^. Some integrins tridimentional structures have been studied and are identified as α1β1, α2β2, α4β1, α5β1, α6β1, αLβ2, αMβ2, αIIbβ3, αVβ3, αVβ5, αVβ6 and α6β4. Most of them have been determined by X-ray crystallography and deposited in the Protein Data Bank^6^. Crystallographic structures meanly of β3 integrin ectodomain fragments have been widely studied to understand the integrin–ligand molecular recognition process^1–7^. In addition, three main integrins α5β1, αvβ3 and αIIbβ3 recognize small peptides containing the Arg-Gly-Asp (RGD)-like molecules in their active site. However, a specific antagonist selective for αV integrins (for example αVβ3/β5) is based on the cyclic peptide (RGD) known ascilengitide^8^. Furthermore, the RGD-peptide can be synthesized either in a cyclic or a linear form, both peptides can enhance cellular integrin-mediated cell adhesion to different surfaces^9^ and help to increase the biocompatibility of the nanoparticules or other materials^10–13^. In recent years, there has been growing interest in describing the binding of specific integrin receptors in chemically different surface biomaterials, which allows controlling the biomolecular interactions between proteins and materials to activate specific signaling pathways. These studies provide very important information for tissue engineering techniques^14^.

On the other hand, in our group we have studied the polypyrrole for its electric conductivity and biocompatibility. Effective use of polypyrrole as biomaterial significantly depends on its electrical properties, topography and surface chemistry composition, among other features^15^. Leveraging its conductivity, a variety of materials with polypyrrole have been developed as supports for cell growth; with the goal of improving cell proliferation. Among other methods, the polypyrrole synthesized by plasma (PPPy) and iodine-doped (PPPy-I) exhibit an extraordinary capacity as culture cell support. Different cell types have been seeded and proliferated on biocompatible scaffolds coated by PPPy^16,17^. One of the most important applications is based on the implementation of mesoparticles of PPPy doped with iodine for spinal cord injury treatment. These particles acted as neuroprotectors and promoted functional recovery in combination with physical therapy. The administration of mesoparticles in rats with spinal cord injury by contusion (TECI), have resulted successful integration in nerve tissue, without exhibiting severe inflammatory response. Additionally, more tissue is preserved; as compared with control subjects with TECI without treatment^18^. Another study using polypyrrole under the same synthesis conditions evaluated the growth of liver carcinoma cells on polylactic acid scaffold surface modified with PPPy. Hepatocyte viability was improved in cultures on modified scaffolds compared with normal scaffolds in the control group; so that the properties of the iodine-doped polypyrrole made it an excellent candidate for the regeneration of liver cells^16^. Due to the proliferation of cell lines and their ability in the regeneration of nerve tissues, iodine-doped polypyrrole synthesized plasma has become a promising biomaterial auxiliary in engineering and in tissue regeneration. However, the mechanism through which the cells are benefiting with this procedure when exposed to the biomaterial is not entirely solved. It is supposed to be due to the wealth of functional groups, as proposed in the structure reported by Kumar *et al*.^19^ and especially to amino groups, but they cannot explain why cells proliferate at a higher rate on scaffolds modified by PPPy, respect to commercial supports containing these amine groups. It is now thought that the wealth of surface groups on synthesized polypyrrole by this method may be the reason for the observed preference, where the polypyrrole is an ideal temporal substitute of extracellular matrix (ECM) necessary for the cell or tissue growth. Our group proposed to analyze the PPPy as if it were a common element of the ECM fibronectin, through which the cells and other components of the ECM are fixed permanently or temporarily. However, whether PPPy might also interact directly with proteins like integrins through amino groups is a question deserving further investigation.

In this work, we performed docking studies in presence of Mg^2+^, to investigate possible molecular recognition between the active site of three different integrins structures (α5β1, αvβ3 and αIIbβ3) and the PPPy structure reported by Kumar *et al*.^19^. This structure has three sites that incorporate different functional groups. We meanly tested –NH_2_ and –OH groups as well as groups proposed by Kumar *et al*.^19^. Our results suggested that when incorporating 1H-azirine, -NH_2_ or –OH group in the PPPy structure, the ΔG_b_ values were higly favorable in the minimized crystallographic structures (α5β1, αvβ3 and αIIbβ3). To study the integrin-PPPy system in more detail, we carried out molecular dynamics simulations by clustering and registering the sensitivity of the ΔG_b_ values to the better coupling obtained. These studies were performed on the integrin α5β1 because it showed the highest ΔG_b_ values when compared with the two integrins. Our results indicated that the ΔG_b_ was very favorable for the integrin α5β1 with the –NH_2_ group PPPy-substituting structure during the 100 ns of molecular dynamic simulation. We observed the establishment of hydrogen bonds with **Asn224** or/and **Asp227** which are important residues of the integrin pocket. The amino group plays a very important role in protein binding, the ΔG_b_ was always favorable during the dynamic calculations with respect to -1H-azirine, –OH and the other functional groups, indicating that the role of the amino group is important in molecular recognition. All these analyses were complemented with IR spectroscopic studies to confirm the functional groups involved in the synthesis of PPPy nanoparticles, and to be able to demonstrate that it could interact directly with proteins provided by the fetal bovine serum for *in vitro* assays, which have been carried out in our laboratory. Furthermore, we are interested in the role of the amino groups present in the polypyrrole structure and its interaction with integrins, mainly in the active site to give a molecular interpretation to the number of experimental studies to the date. This study provides new insights at the molecular level on the interaction of PPPy nanoparticles with integrins because this protein can recognize a great variety of RGD-containing ligands as well as biomaterials.

## Results and Discussion

### Nanoparticles characterization

**Figure 1:**
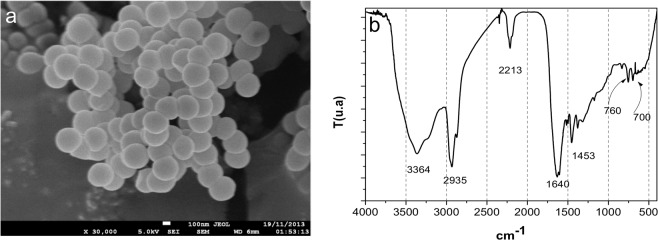
(**a**) Microphotograph and (**b**) Infrared spectrum of the nanoparticles of PPPy.

Figure 1a shows the SEM image of the PPPy nanoparticles which average size is around 140 nm. They form aggregates and were dispersed by ultrasonic pulses. The nanoparticles synthesized by plasma polymerization were characterized by Fourier Transform Infrared Spectroscopy with an Attenuated Total Reflectance unit, FTIR-ATR Perkin Elmer GX System with an ATR unit Smith Diamond Durasample II. The Fig. 1b depicts the FTIR spectrum, and the peaks widths obtained are characteristic of plasma synthesized materials. Additionally, in the region of 3500–3300 cm^−1^ a broad band is observed which can be assigned to the asymmetric and symmetrical stretching vibrations of the -NH or -NH_2_ groups. This band appears at 3364 cm^−1^ in the PPPy spectrum. In the range of 2960–2872 cm^−1^ there are two characteristic bands of the -CH groups. The frequency 2935 cm^−1^ can be assigned to the asymmetrical stretching mode (νasCH). The second close frequency at 2800 cm^−1^ can be attributed to the symmetrical stretching vibrations of this group (νasCH). In the frequency range 2260–2220 cm^−1^ a low intensity band is present, which can be assigned to the stretching vibrations of the C≡N (nitrile) and C≡C bond. Nitriles are characterized by a weak to medium absorption, which appears in 2213 cm^−1^ of this spectrum. In this same region we observed the stretching band characteristic of disubstituted acetylenes with different groups (-C≡C-). The low intensity of the acetylenes signal is due to the symmetry of multiple bonds, if their substituents are the same, we do not observe the stretching band of this group in the IR spectrum. So we can consider that the intensity of the band at 2213 cm^−1^ has contributions from the vibration stretching frequencies of the nitrile and acetylene groups. The presence of the C-H, C≡C and C≡N groups is caused by the disruption of some aromatic pyrrole rings due to the high energy of the plasma discharge, which also causes the fragments to be dehydrogenated.

The strong signal at 1640 cm^−1^ in Fig. 1b can be attributed to the bending vibration of the N-H bond of amides (H-N-C=O) or amines (-NH, -NH_2_), to the stretching vibration of the C bond = C of alkenes and to the stretching vibration of the bond C=N of imides (RCH = NR). These fragments may also be formed by the destruction of the pyrrole rings during the plasma polymerization process. In the 1580–1400 cm^−1^ region vibrations of the aromatic polynuclear skeleton, involving carbon-carbon stretching vibrations within the ring, are present. It can be suggested that the signals at 1580 cm^−1^ and 1453 cm^−1^ correspond to vibrations in the plane of the groups C=C and C-H in the polypyrrole rings.

The low intensity band near 1300 cm^−1^ can be assigned to the stretching vibration of the C-N bond of the aromatic ring, this absorption appears at higher frequencies due to the resonance of the pyrrole ring. The band at 760 cm^−1^ is attributed to the vibration of the skeleton and is indicative of the formation of polymer chains. The band appearing at 700 cm^−1^ in the pyrrole spectrum can be attributed to the bending vibration of the methylene group (H2C:).

### Cells-nanoparticles interaction

**Figure 2:**
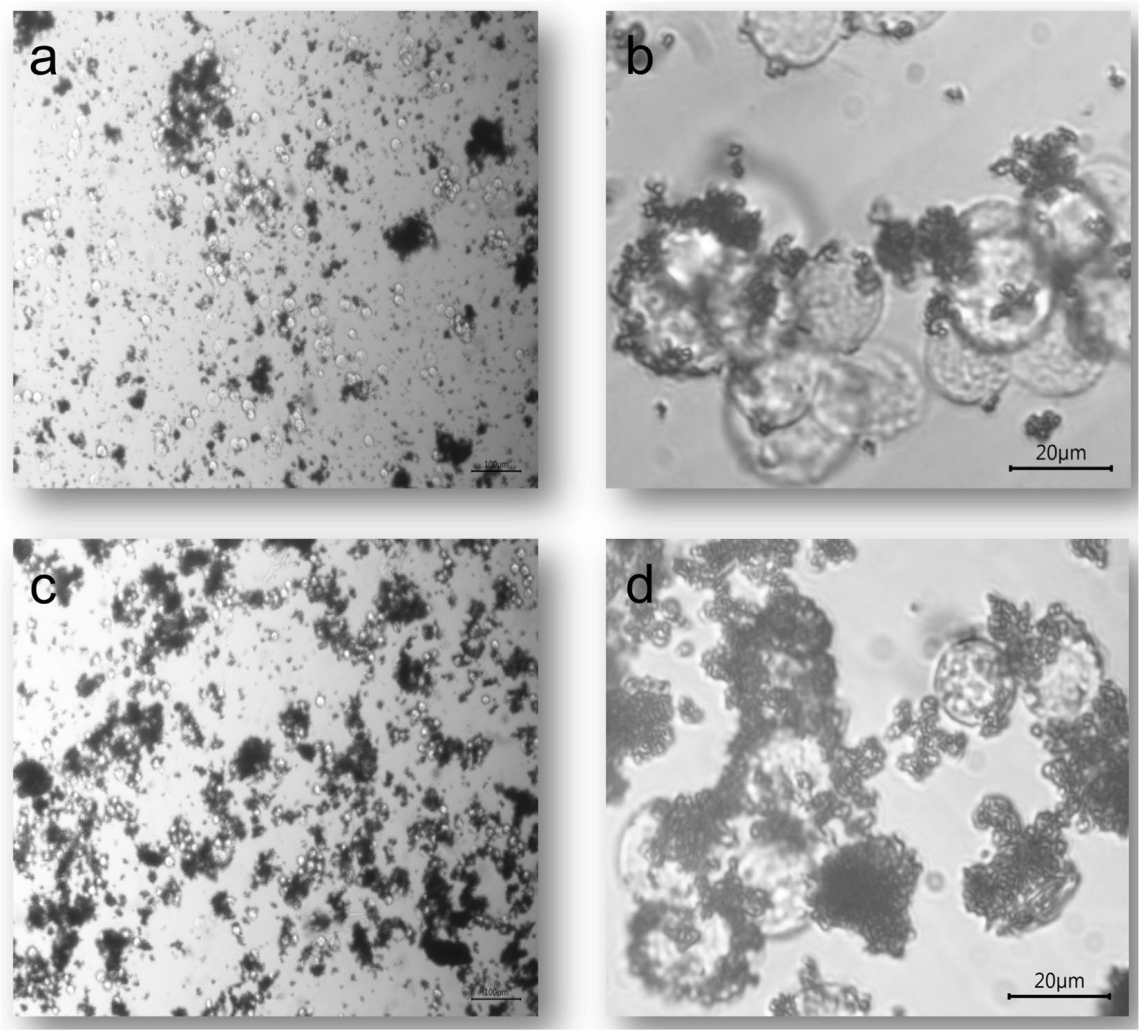
Optical Micrographs of cell cultures, (**a**) first day without serum (X100) (**b**) first day without serum(X400) (**c**) first day with serum (X100) (**d**) fifth day with serum(X400).

In order to study the nanoparticles and cells interaction two types of cell cultures were prepared, one just containing PBS to avoid the presence of proteins and study the direct Cell-PPPy interaction, and the other with bovine fetal serum. Figure 2 shows the optical micrographs on the first day of culture, at two optical microscope amplifications. Figure 2a,b show the culture in PBS, in this case, there are some cells fixed to the PPPy aggregates. In Fig. 2c,d the culture with fetal bovine serum is showed, it is clear that in this case the PPPy-cells aggregates are larger and there are almost no cells without PPPy, which is an evidence of an effective intermolecular interaction.

### Computational determinations: binding energy

#### Molecular docking studies

**Figure 3:**
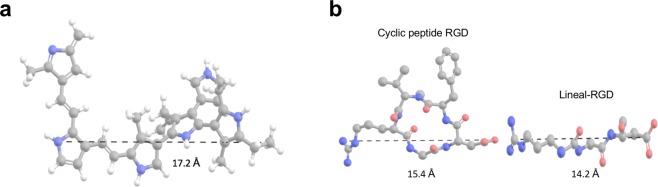
Chemical structures. (**a**) PPPy structure indicating the length. (**b**) Cilengitide structures: cyclic peptide and lineal RGD. The lengths were determined with the Maestro program^21^.

To understand the molecular interaction between integrins and PPPy, we made docking simulations for every integrin type studied in this work. Our studies suggest that the residues of aspartate are important for the binding affinity of PPPy molecule to integrins because of their negative charge, and that residues of serine and glutamate make contact with the PPPy trough magnesium ion. This is consistent with the three crystallographic structures of integrins complexed with RGD that have been recently determined by some authors^2,7,20^ and used in this work. They show that charge-charge interactions play an important role in the integrin-ligand binding, from which aspartates and glutamates are key for recognition process. Besides, it can be see that RGD cyclic has a similar chemical structure and slightly smaller to the PPPy molecule (Fig. 3a). Figure 3b shows RGD-peptide in its cyclic and linear form. Therefore, the question of whether PPPy molecule may directly interact with integrins, and how this event contributes to promote PPPy as an element ideal to study the interactions that the ECM has allowing the growth and bonding of the cell, which is the matter of interest in this study.

**Figure 4:**
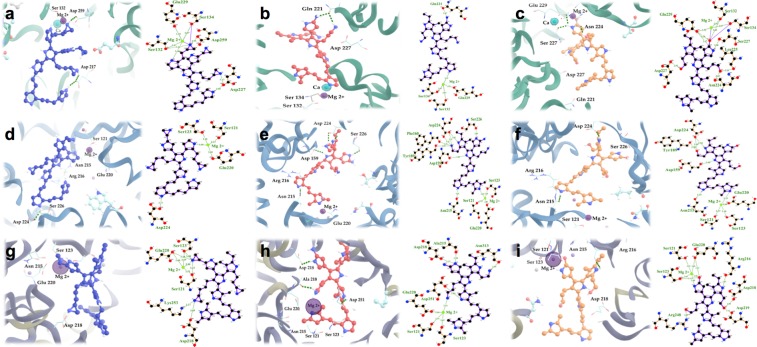
Structures of the highest scoring conformers of PPPy with different functional groups obtained by docking studies on the binding site with tree different integrins. For α5β1 integrin the three more favorable complexes according to ΔG_b_ were: (**a**) PPPy-aziridine, (**b**) PPPy-NH_2_ and (**c**) PPPy-OH. In the case αIIbβ3 were: (**d**) PPPy-aziridine, (**e**) PPPy-NH_2_ and (**f**) PPPy-OH too. For αvβ3 integrin the more favorable complexes were: (**g**) PPPy-aziridine, (**h**) PPPy-NH_2_ and (**i**) PPPy-OH. The 2D models are shown, depicting the interaction of the different integrins with PPPy-functional groups were obtained with LIGPLOT + program^22^. The amino acids and hydrogen bonds were mapped on each 3D structure using Visual Molecular Dynamics (VMD) 1.9.1 program^23^.

Furthermore, we performed a detailed analysis of these molecules in 3D structures and made 2D diagrams of the interaction between integrins in presence of Mg^2+^and PPPy for the most favorable complexes in each system (Fig. 4). Docking results revealed that the integrin α5β1 (3VI4) interacts with PPPy (by means of aziridine group in the three sites in Fig. 4a, -NH_2_ group in Fig. 4b, and –OH group in Fig. 4c) involving mainly Glu229, Ser124, Ser132, that interact with the Mg^2+^, and **Asp227** that match with reported in the literature^7^ as well as **Asn224**. Figure 4d–f show docking results of PPPy structure (aziridine, -NH_2_ and –OH respectively) with magnesium-bound αIIbβ3 (2VDR), involving some residues that constitute the binding site, like Ser121, Tyr122, Ser123, Tyr189, Asn215, Arg216, Glu220 and **Asp224**^20^. Finally, for αvβ3 (1L5G) with PPPy structure (aziridine, -NH_2_ and –OH) docking results are shown in Fig. 4g–i, respectively. These complexes involved mainly the amino acids of the binding site: Ser121, Ser123, Glu220 showing contacts with Mg^2+^, and **Asp218**^2^. In other words, the residues above mentioned coincide with the amino acids shown in Fig. 8 for each system analyzed here. These docking results allowed us to further determine the polar/electrostatic (ΔG_elec_) and non-electrostatic (ΔG_non-elec_) contribution to the free energy of binding (ΔG_b_) in the integrins-PPPy film interaction for the best pose, in each case.

### Binding energy: electrostatic and non-electrostatic determinations

In Table 1 shows a list of the ΔG_b_ values for each system integrin-PPPy obtained. Comparison of the binding energies shows that the complexes α5β1 had very favorable electrostatic contributions (except acetylene and nitrile) respect to αIIbβ3 and αvβ3. The interaction of the three integrins with PPPy-aziridine, (the functional group proposed by Kumar *et al*.^19^) was more favorable than the substitutions with -NH_2_ and -OH group in the PPPy structure. In most cases, the binding energies of the complexes are driven by electrostatic interactions, where the direct Coulombic was the most important contributor, over the hydrophobic interactions (except 1-propene, acetylene and nitrile).

**Table 1:** Binding energies (ΔG_b_) along with their contributors: solvatation energy (*Δ*G_solv_), Coulombic energy (*Δ*G_coul_), and non-electrostatic energy (*Δ*G_non-elec_) determined at pH 7.0 by APBS^24^ and VMD 1.9.1^23^ respectively.

PPPy-X^a^	Integrin type^b^	ΔG_solv_ (kJ/mol)	ΔG_coul_ (kJ/mol)	ΔG_non-elec_ (kJ/mol)	ΔG_b_^c^ (kJ/mol)
	α5β1	22	−1758	−18	−1754
	αIIbβ3	28	−395	−20	−387
	αvβ3	28	−30	−25	−27
	α5β1	23	−453	−17	−447
	αIIbβ3	23	−205	−24	−206
	αvβ3	39	−28	−23	−12
	α5β1	23	−74	−36	−87
	αIIbβ3	14	−40	−22	−48
	αvβ3	20	−7	−21	−8
	α5β1	24	−18	−22	−16
	αIIbβ3	11	−24	−23	−36
	αvβ3	22	−3	−25	−6
	α5β1	23	−6	−19	−2
	αIIbβ3	−23	23	−26	−26
	αvβ3	33	−11	−24	−2
	α5β1	27	0.1	−18	9
	αIIbβ3	28	−6	−24	−2
	αvβ3	25	4	−24	5
	α5β1	24	6	−18	12
	αIIbβ3	19	22	−23	18
	αvβ3	22	79	−24	77

^a^PPPy**-**X, where X represents the substitution of functional groups mentioned in this Table.

^b^Crystallographic structures of the integrins were minimized (see Materials and Methods section) ^c^Binding energy (ΔG_b_) as given by Eq. (2).

**Figure 5:**
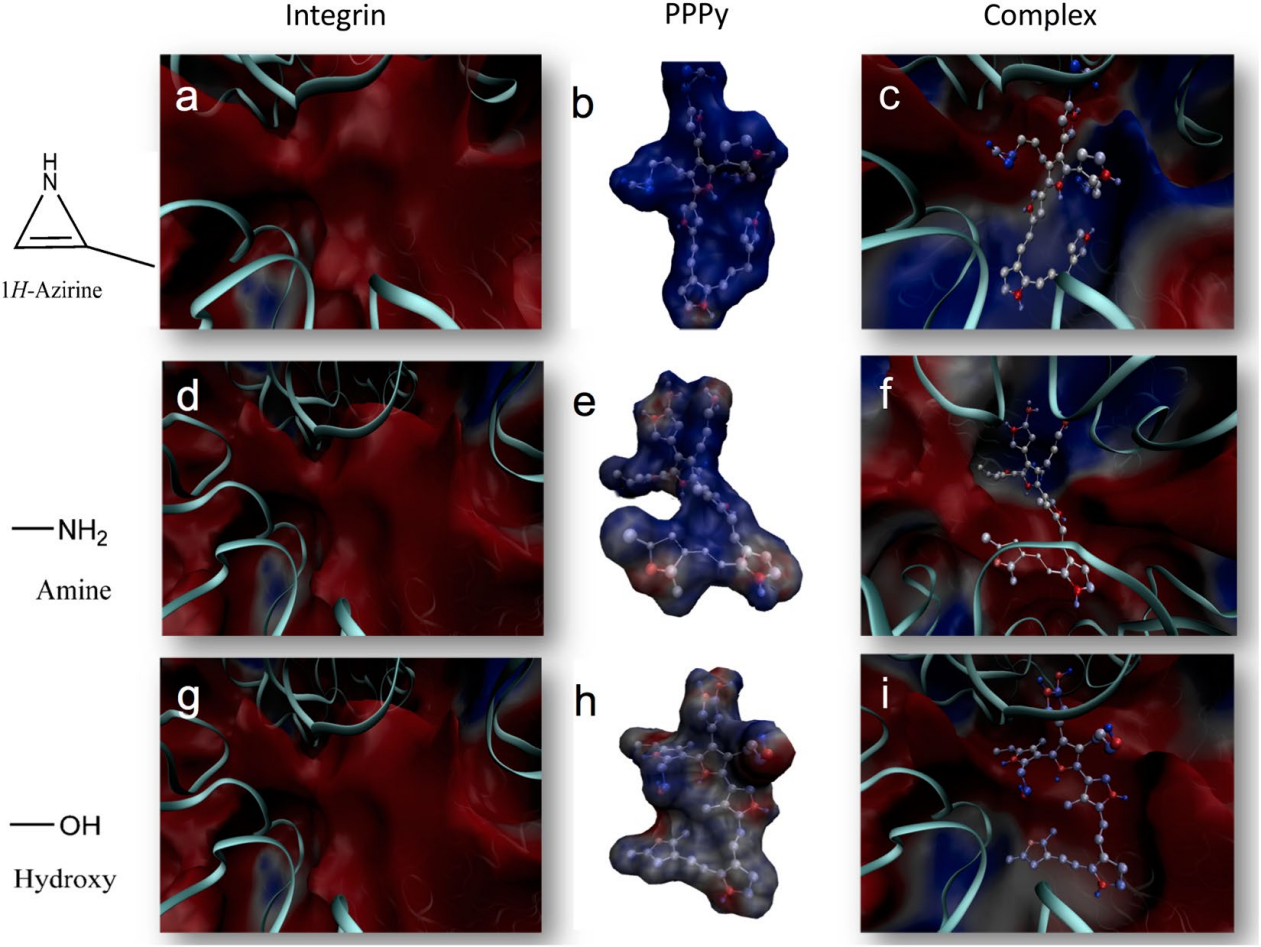
Electrostatic potentials of the free integrin (**a,d,g**), free ligand (**b,e,h**) and the complex formed for α5β1-PPPy-functional groups like aziridine (**c**), –NH_2_ (**f**) and –OH (**i**).

Figure 5 depicts the electrostatic profiles of the free integrin α5β1 (a, d and g) with best binding poses aziridine (b), -NH_2_ (e) and –OH (h). The PPPy-aziridine bound to integrin α5β1 (Fig. 5c) suggests the predominance of the contribution of the electrostatic interactions to the binding process is very strong mainly via the negative charge of the carboxyl group of Asp227 that interacts with the N-H moiety of aziridine and with a nitrogen atom of PPPy template. We also observed the formation of hydrogen bonds. For the integrin α5β1 bound to PPPy-NH_2_ (Fig. 5f) electrostatic interactions are established between the Oε1 atom of Gln 221 and the nitrogen atom of -NH_2_ group. Finally, for the integrin α5β1 to PPPy-OH interaction (Fig. 5i) the electrostatic potential is less favorable because these –OH groups subtituided on the PPPy template form hydrogen bonds with carboxyl group of Asp 227 (OH┈O^−^ respectively) creating a weak repulsive environment.

### Molecular dynamics simulations

To have more detail information about the integrin-PPPy interaction, we carried out extensive molecular dynamics simulations and docking studies mainly for integrin α5β1, because this protein yielded the highest ΔG_b_ interaction values when interacting with the PPPy structures (Table 1). We simulated 100 ns of molecular dynamics to analyze conformational changes by clustering. These dynamic calcultations allowed us to focus docking studies and to better evaluate specific electrostatic contributions to ΔG_b_ at 46070 and 96470 ps (named as cluster1 and cluster2 from now on, respectively) along the trajectory. Correspondent values are shown in Table 2.

**Table 2:** ΔG_b_ summary of the integrin α5β1with PPPy at different times during the simulation trajectories.

Integrin α5β1-PPPy-X		ΔG_solv_ (kJ/mol)	ΔG_coul_ (kJ/mol)	ΔG_non-elec_ (kJ/mol)	ΔG_b_^a^ (kJ/mol)
	cluster1	88	−313	−25	−251
	cluster2	−6	−60	−24	−90
	cluster1	72	−11	−25	36
	cluster2	116	−19	−24	72
	cluster1	92	1	−25	68
	cluster2	134	4	−25	113
	cluster1	58	−7	−24	27
	cluster2	126	−7	−24	95
	cluster1	76	3	−24	54
	cluster2	51	−2	−24	25
	cluster1	12	−1	−24	−13
	cluster2	140	−0.1	−23	−116.9
	cluster1	63	−8	−24	31
	cluster2	117	−2	−23	91

^a^ΔG_b_ as given by Eq. (2).

The analysis of the binding energies of Table 2 shows that integrin α5β1-PPPy-NH_2_ system was very favorable in the different trajectories and that the process is driven by electrostatic interactions, which supports our experimental studies on the role of the amino group in the interaction of PPPy with proteins. In the rest of the complexes, the ΔG_b_ values are less favorable, since more energy is required to solvate the complexes, however the non-electrostatic component was favorable with respect to the electrostatic contribution, mainly in the case of integrin α5β1-PPPy-1-propene. All these trajectories show important conformational changes during the molecular dynamics simulations and this is reflected in the ΔG_b_ values, except for the integrin α5β1-PPPy-NH_2_ complexes.

The PPPy-NH_2_ structure establishes hydrogen bonds mainly with Gln 221, Ser 132, Ser 134 and **Asp 227** in the binding site of the minimized integrin α5β1 shown in Fig. 4b and getting a ΔG_b_ = −447 kJ/mol (Table 1). At cluster1, the residues that participated in the binding site, mainly were Tyr 296, **Asn 224** and Asp 267 with a ΔG_b_ = −251 kJ/mol. Finally, the amino acids that established hydrogen bonds were Ser 227, Ser 229, Lys 254, Phe 187 and Asp 226 with PPPy-NH_2_ at cluster2 and was obtained a ΔG_b_ = −90 kJ/mol. The integrine α5β1-PPPy-NH_2_ complexes established hydrogen bonds with **Asn 224** and/or **Asp 227**, which are important residues of the active site. However, we also found differences in the trajectory at cluster2, because the PPPy-NH_2_ structure interacts with residues near to the active site (5 Å) as an aspartate and serines; wich are similar residues to the binding pocket of the integrin α5β1.

**Figure 6:**
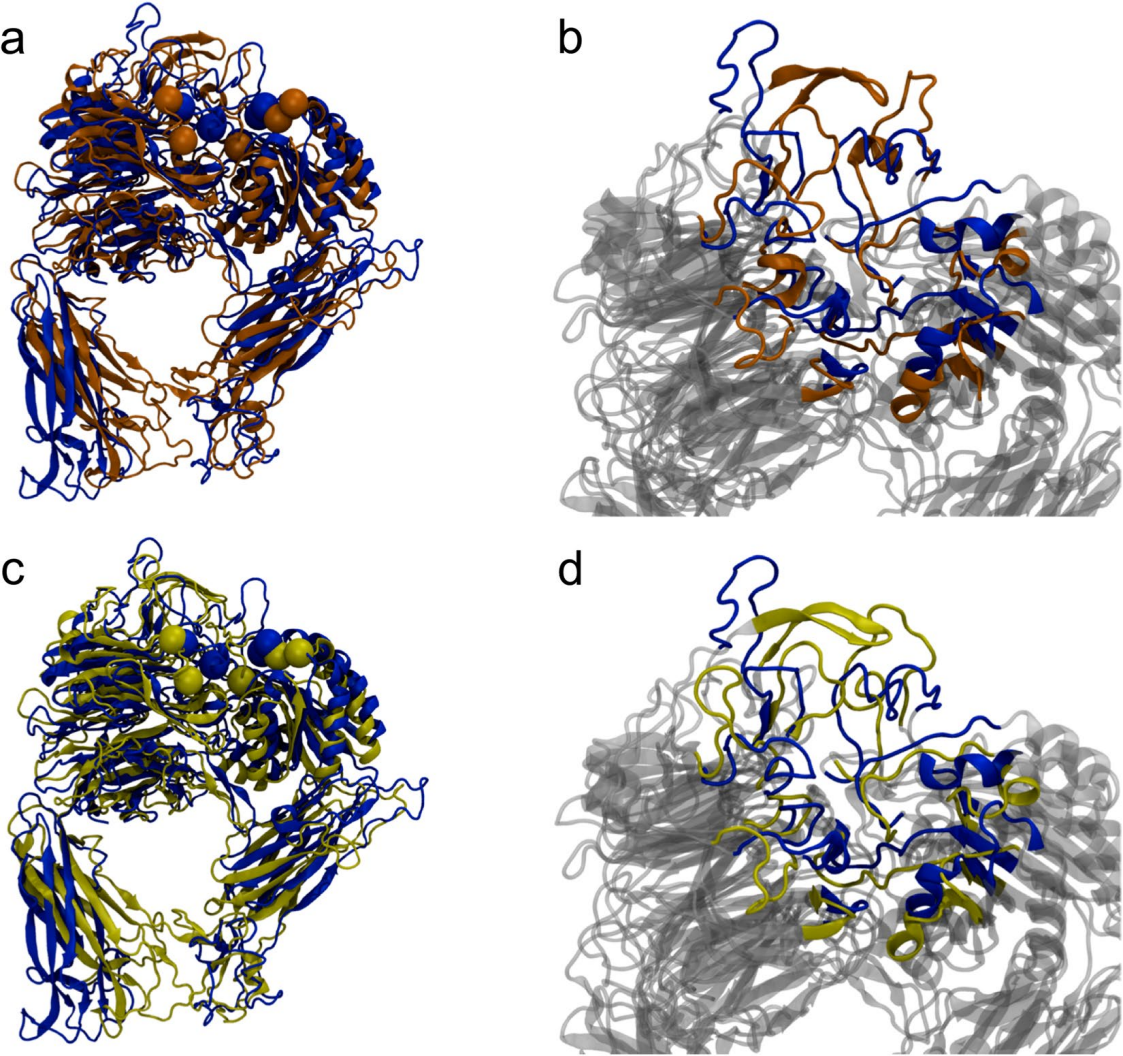
Superposition of the 3VI4 structure (blue) with respect to trajectory cluster1 (brown) and cluster2 (yellow). (**a**) Minimized structure of 3VI4 vs cluster1, and (**b**) the respective zoom of loops. (**c**) Minimized structure 3VI4 vs cluster2 and (**d**) the respective zoom. The residues that are part of the active site and are highlighted in spheres.

Figure 6 depicts the superposition of the 3VI4 structure (blue color) with two of its conformations along the molecular dynamics trajectory; cluster1 (brown color) and cluster2 (yellow color). A zoom of the loops is shown in Fig. 6b,d respectively. This figure clearly shows slightly fluctuations of the binding site during the simulation (Fig. 6a,c). Also, the comparison of the superposition of initial docking results and the clusters structures indicated that the loops near the active site are flexible during the simulation time (Fig. 6b,d).

## Conclusions

The present study confirmed previous findings of our group on the role played for the amine functional groups of the PPPy, as ideal temporal substituents to promote the cell interaction of ECM for tissue engineering. We suggest for the first time, that the interaction of PPPy could be also occurring at a molecular level, since our computational models and *in vitro* assays indicate that the interactions that lead to the binding of PPPy with various proteins such as integrins are mainly due to Coulombic interactions, an important term in the electrostatic component, and in minor proportion the non-electrostatic components. However, the molecular dynamics simulations showed slightly fluctuations of integrin α5β1 joined to PPPy-NH_2_, in most trajectory frames hydrogen bonds were established with **Asn 224** and/or **Asp 227** except on cluster2 structure. It is important to note that the ΔG_b_ value for the integrin-PPPy-NH_2_ system was always favorable before and during the molecular dynamics simulations. In general, the interactions obtained are in accordance with the *in vivo* experiments and they will require further detailed study, but meanwhile, they represent an alternative and very reliable explanation for the surface activity of the biomaterial. Computational models provide a qualitative understanding of the driving forces responsible for the binding of integrins to PPPy through which the cells and other components of the ECM can improve cell proliferation.

## Materials and Methods

### Plasma polymerization of pyrrole nanoparticle

The plasma reactor is described in a previous work^17^, is formed of a pyrex glass tube of 25 cm length, 9 cm of external diameter and 5 mm thickness, each end of the tube is sealed by a stainless steel lids and in each lid there are two access ports and one central access, where one electrode for plasma discharge is introduced. In a lid and using the access ports, a pirani probe (Edwards) is placed to measure the pressure inside the discharge chamber, by the other access a vacuum system is connected. In the opposite lid, one of the ports is used to introduce the pyrrole monomer into the reaction chamber. The vacuum system consists of a mechanical pump and a cold trap by particles. The electrodes that are introduced to the polymerization reactor are made of stainless steel and have a diameter of 7 cm. These electrodes are connected to a CESAR model 1500 source, 40 W power is applied to a RF of 13.56 MHz, the pressure into reactors was of 2 Torr and the distance between electrodes was of 5 cm.

### Culture of the NG 108-15 cell line with the polypyrrole nanoparticles

Before the cell culture, the NG 108-15 cells were washed tree times with phosphate buffered saline (PBS) for five minutes to remove the residues of media culture. Cultures were divided into two groups, one using PBS as culture medium and the other with Dulbecco´s Modified Eagle Medium (DMEM) supplemented with 10% fetal bovine serum and 1% of L-Glutamine. Around 1 × 106 NG 108-15 cells ware deposited in a 15 ml falcon tube with 2 ml of culture medium and then 200 μg of PPPy nanoparticles were added. The cells with the PPPy nanoparticles were centrifuged at 1200 rpm for 5 minutes, the pellet formed was lightly resuspended and placed in 35 × 10 mm Petri dishes to be incubated for one hour at 37 °C under an atmosphere of 5% CO_2_ −95% air. After one hour, the cultures were observed under an optical microscope.

### Integrins and plasma-polymerized pyrrole (PPPy) structures

**Figure 7:**
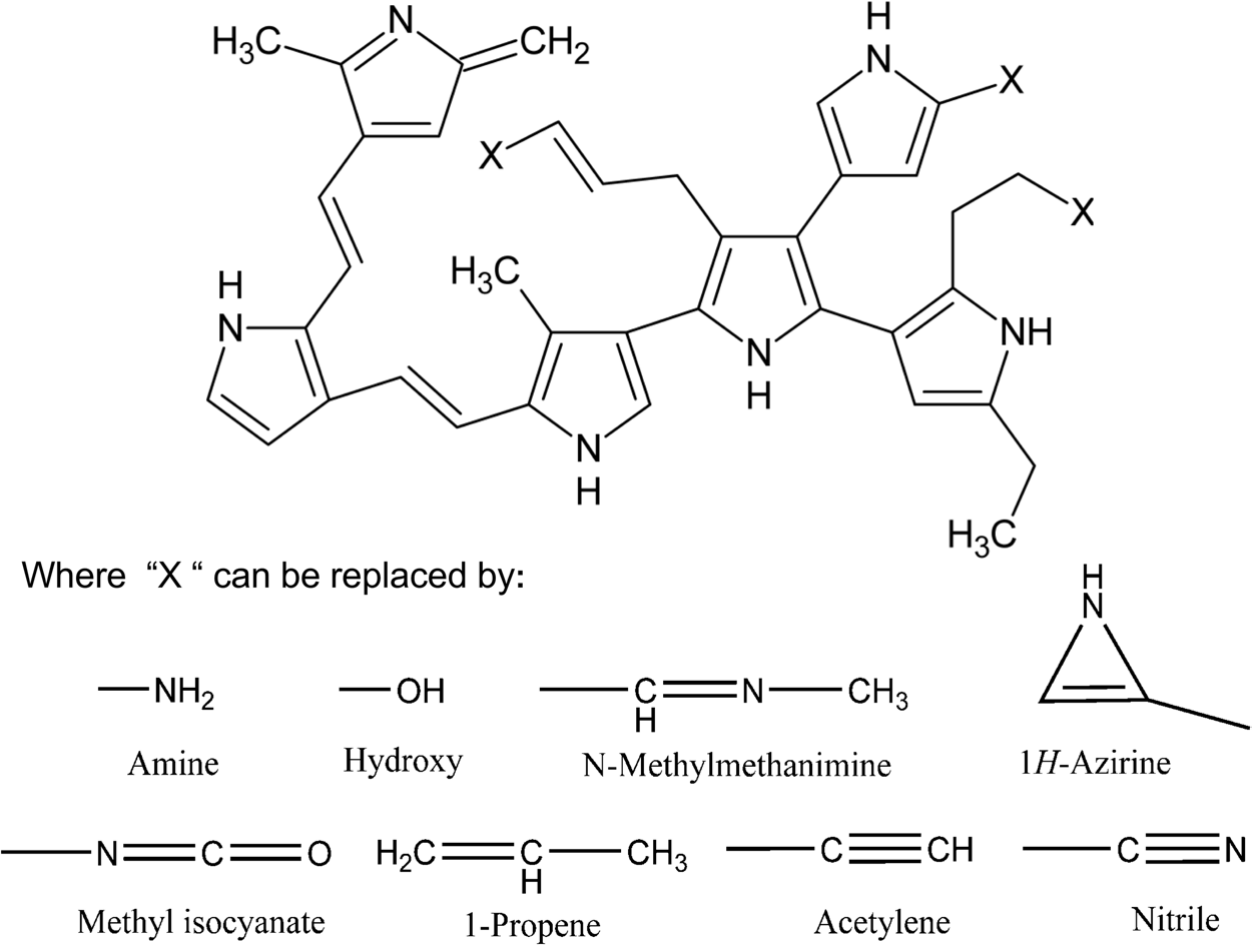
PPPy structure was proposed by Kumar *et al*.^19^. Each functional group was replaced in the three “X”. The template structure and functional groups were built with the Gaussian09 program^27^.

Specific recognition of RGD peptide by three different classes of integrins: α5β1 (PDB ID 3VI4) in its complexed form^7^, αvβ3 (PDB ID 1L5G)^2^ and αIIbβ3 (PDB ID 2VDR)^20^ were used in presence of Mg^2+^. Integrins structures were briefly energy-minimized with 100 steps of the steepest-descent method in CHARMM38b2^25^, with the CHARMM36^26^ potential, to relieve remaining steric clashes. In Fig. 7 depicts the chemical structure proposed for the plasma-polymerized pyrrole (PPPy) film including some functional groups reported by Kumar*et al*.^19^. Our group has recently described preliminary evidence of the involvement of amine and hydroxyl groups in the conduction mechanism in the polymer film. We constructed this structure where each functional group was added to this template including –NH_2_ and –OH groups using Gaussian09 program^27^.

### Computational docking: Interaction between integrin-PPPy complexes

Integrins and PPPy as described above were employed for docking assays. We modeled two conditions by docking: the possible binding on the whole receptor (data not shown), and in the pocket binding of integrins based in the crystallographic structures complexed with RGD. The amino acids of α5β1 (PDB ID 3VI4)^7^ were Ser132, Ser134, **Gln 221**, Glu229, **Asp 227**. For αvβ3 (PDB ID 1L5G)^2^ amino acids were Ser121, Ser123, Asn215, **Asp218** and Glu220. The residues of αIIbβ3 (PDB ID 2VDR)^20^ were used were Ser121, Tyr122, Ser123, Tyr189, Asn215, Arg216, Glu220 and **Asp224**. All dockings were performed with magnesium-bound integrins. Docking was performed using the program Autodock Vina^28^, and the docking conditions were set in Pymol^29^. The integrins were kept fixed, and the PPPy had freely rotating bonds. Approximately 1000 docking attempts were carried out for each system, keeping for each attempt only the best binding pose. Likewise, for the integrin α5β1 with PPPy we carried out docking studies in the trajectories generated by clustering during 100 ns. For detailed analysis of the interactions at binding site, we chose the pose with the best binding energy and/or the most extensive protein contacts in each case. Also integrins residues that form hydrogen bonds were highlighted with LIGPLOT + program^22^

### Comparison between RGD-bound and RGD-docked in the active site

**Figure 8:**
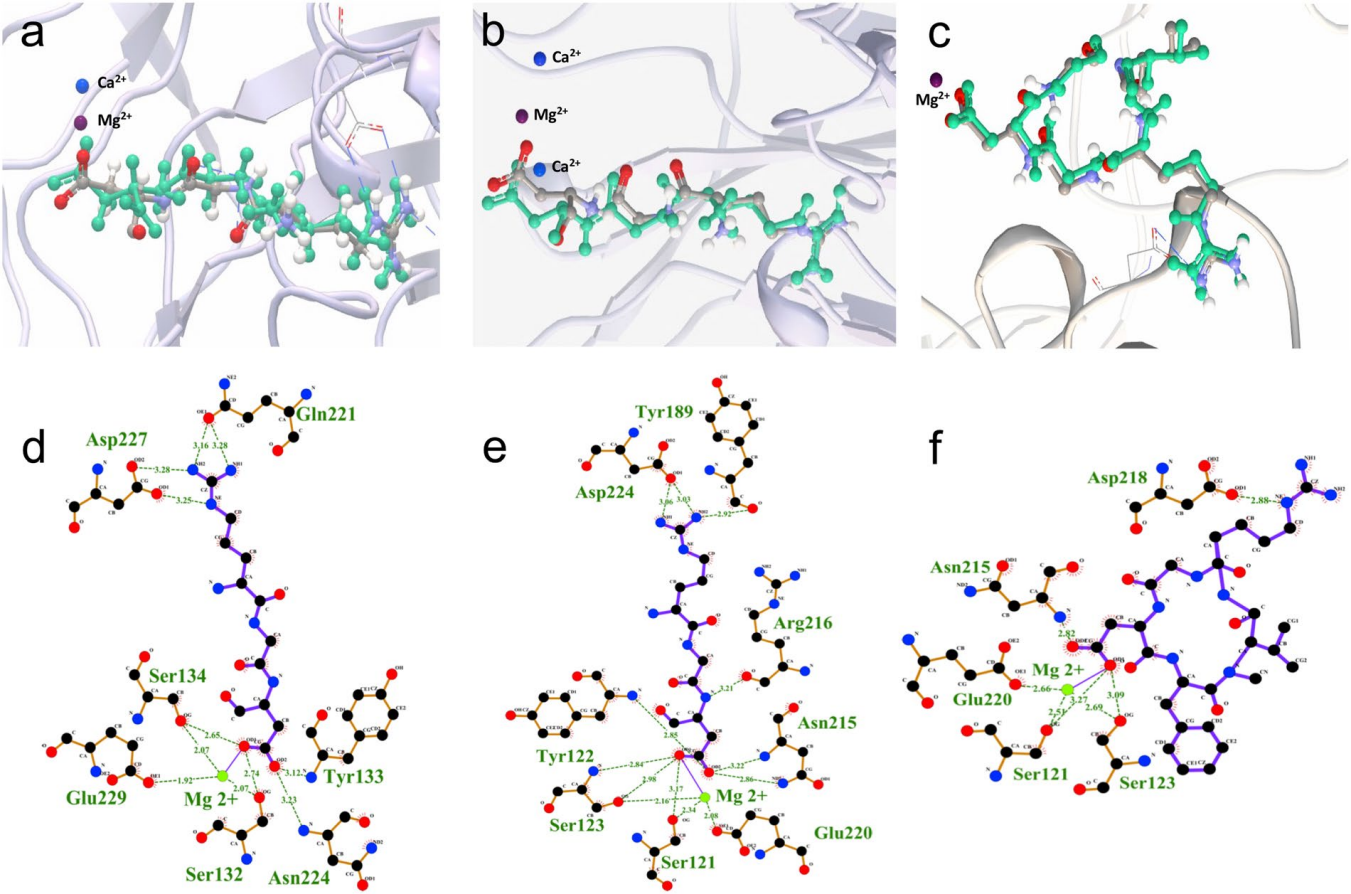
Comparison between each crystallographic RGD molecule and the best pose obtained by AutodockVina docking software^28^ (green) in the binding pocket. (**a**) α5β1 (PDB ID 3VI4)^7^. (**b**) αIIbβ3 (PDB ID 2VDR)^20^ and (**c**) αvβ3 (PDB ID 1L5G)^2^. The amino acids and hydrogen bonds involved mainly in the binding for each crystallographic integrin were highlighted and determined by LIGPLOT + program^22^.

To validate our studies, we performed docking assays on minimized crystallographic structures complexed with RGD (α5β1, αvβ3 and αIIbβ3). Docking procedure was the same as described above. Figure 8 depicts the overlap between the minimized crystallographic and docked RGD of α5β1 (a), αIIbβ3 (b) and αvβ3 (c) as well as hydrogen bonds (d, e and f respectively).

### Molecular dynamics simulations

The stability of the integrin α5β1^7^ was studied by molecular dynamics simulations for 100 ns. 3VI4 (PDB code) were used as initial coordinates. Charmm-gui web server^30^ (www.charmm-gui.org) was used to solvate (10 Å cubic box around the protein) and ionized (enough ions to neutralized the protein) the initial structure. Molecular dynamics simulations were using GROMACS^31,32^ with charmm36 potential^26^. All parameters were used as suggested by the charm-gui web server. Clustering of the final trajectory was done using Gromacs utilities over backbone atoms using 2.5 Å cutoff between each cluster. Likewise, for the integrin α5β1with PPPy we carried out docking studies (∼1000 attempts) in each trajectories generated by clustering during 100 ns.

### Binding energy and electrostatic profiles

The binding energy for each complex was calculated considering the electrostatic/polar and non-electrostatic/non-polar contributions. The general method for calculating the electrostatic energy is divided in two components: mainly solvation and coulombic, as described by Baker *et al*.^24^ and given by:

1 $${{\rm{\Delta }}{\rm{G}}}_{{\rm{elec}}}={{\rm{\Delta }}{\rm{G}}}_{{\rm{solv}}}+{{\rm{\Delta }}{\rm{G}}}_{{\rm{coul}}}$$

where ΔG_solv_ represents the computational determination for solvation energies and ΔG_coul_ represents coulombic energies of integrin-PPPy complexes, which were calculated using the Adaptive Poisson-Boltzmann Solver (APBS) program^24^. We followed the same protocol as in our previous reports^33–37^. Dielectric constants of 78 and 4 were used for water and protein, respectively. Parameters were taken from the PDB2PQR server^38^. Ionic radii and atomic charges were assigned from the forcefield CHARMM^39^. PROPKA was used to assign the protonation state of ionizable residues at pH 7.0^40^. PPPy atomic charges were assigned from the force field implemented in the AutodockVina program^28^. The non-electrostatic contribution to the binding energy was estimated by multiplying the change in solvent accessible surface (ΔASA) upon binding by coefficient γ, which is an interfacial tension of 5 cal•mol^−1^ Å^−2^ ^41^. The calculations for ASA were done using theVisual Molecular Dynamics (VMD) 1.9.1 program^23^, implying a probe radius of 1.4 Å. The final binding energy calculated (ΔG_b_) was given by:

2 $${{\rm{\Delta }}{\rm{G}}}_{{\rm{b}}}={\rm{\Delta }}{{\rm{G}}}_{{\rm{s}}{\rm{o}}{\rm{l}}{\rm{v}}}+{{\rm{\Delta }}{\rm{G}}}_{{\rm{coul}}}+{{\rm{\Delta }}{\rm{G}}}_{{\rm{non}} \mbox{-} {\rm{elec}}}$$

where ΔG_non-elec_ represents the non-electrostatic energy.

The electrostatic profiles of the complex and free species were determined using the command pot at the input of APBS calculations at pH 7.0 with the CHARMM parameters (using the parameters mentioned above)^24,39^. The results of these electrostatic potential calculations were visualized with VMD program^23^.
